# Real-World Lenvatinib *Versus* Sorafenib in Patients With Advanced Hepatocellular Carcinoma: A Propensity Score Matching Analysis

**DOI:** 10.3389/fonc.2021.737767

**Published:** 2021-10-25

**Authors:** Yuan-Hung Kuo, Sheng-Nan Lu, Yen-Yang Chen, Kwong-Ming Kee, Yi-Hao Yen, Chao-Hung Hung, Tsung-Hui Hu, Chien-Hung Chen, Jing-Houng Wang

**Affiliations:** ^1^ Division of Hepatogastroenterology, Department of Internal Medicine, Kaohsiung Chang Gung Memorial Hospital and Chang Gung University College of Medicine, Kaohsiung, Taiwan; ^2^ Division of Hematology-Oncology, Department of Internal Medicine, Kaohsiung Chang Gung Memorial Hospital and Chang Gung University College of Medicine, Kaohsiung, Taiwan

**Keywords:** hepatocellular carcinoma, lenvatinib, propensity score (PS) matching (PSM), sorafenib, progression-free survival

## Abstract

**Background:**

Lenvatinib is approved for patients with advanced hepatocellular carcinoma (HCC) due to its non-inferiority to sorafenib of overall survival (OR) in clinical trials. This study was to compare the effectiveness and safety of lenvatinib and sorafenib in the real world.

**Methods:**

We retrospectively evaluated 338 patients with unresectable HCC who had undergone lenvatinib or sorafenib treatment between January 2018 and August 2020. Propensity-score matching analysis was performed with a 1:2 ratio to reduce the real-life baseline difference between the two groups.

**Results:**

A total of 210 patients (Male/Female: 150/60, mean age: 65.8 years) were recruited including 70 patients in the Lenvatinib group and 140 patients in the Sorafenib group. Compared with sorafenib, lenvatinib had significantly longer progression-free survival (PFS) (5.2 *vs* 3.3 months, p=0.019) but similar OR (13.3 *vs* 11.8 months, p=0.714). Additionally, lenvatinib had better disease control rates (62.3 *vs* 48.6%, p=0.029) and equivalent incidences of treatment-related adverse events over sorafenib. In multivariate analysis, lenvatinib was associated with better PFS over sorafenib (hazard ratio: 0.49, 95% confidence interval: 0.3–0.79, p=0.004) after adjustments of albumin-bilirubin grade and alpha-fetoprotein level; however, different agents using lenvatinib or sorafenib did not contribute to OS, whether in univariate or multivariate analysis. Patients who failed lenvatinib had a lower proportion of having sequential systemic therapies compared with the Sorafenib group (36.2 *vs* 47.8%, p=0.02). The most frequently used sequential therapy following lenvatinib and sorafenib was chemotherapy (n=9, 42.8%) and regorafenib (n=33, 50.8%), respectively.

**Conclusions:**

In clinical real-life practice, lenvatinib illustrated promising survival benefits and acceptable safety for patients with unresectable HCC, while reducing the risk of progression disease compared with sorafenib. Additionally, lack of approved post-lenvatinib systemic therapies is a serious issue in the real world.

## Introduction

Hepatocellular carcinoma (HCC) is the most common liver cancer and one of the leading causes of cancer-related deaths worldwide, accounting for approximately 700,000 mortalities per year (1). HCC at early stage can often be curatively treated by effective options such as surgical resection, liver transplantation, and local ablation, which could lead to better survival outcome (2). On the contrary, advanced-stage HCC frequently presents with poor prognosis because of limited potential treatment modalities offered (3). Despite the improvement of HCC diagnostic measures and screening systems, many patients are still diagnosed at an advanced stage where systemic therapy is the main recommended treatment option (4–6). Sorafenib, the first approved agent for patients with advanced HCC in 2007, is a multikinase inhibitor that can target several protein receptors (VEGFR, PDGFR, KIT, and RET) to block vascular angiogenesis as well as inhibit several cell signaling pathways (Raf-1, B-Raf, and Ras/Raf/MEK/ERK) to impair tumor proliferation (7). Approval of sorafenib is based on two randomized, open-label, phase III clinical trials, SHARP study and AP study, where sorafenib significantly improved overall survival (OS) in patients with advanced HCC compared with a placebo (8, 9). However, the development of other first-line systemic therapies for advanced HCC was dismaying, until 2018, when lenvatinib first demonstrated its therapeutic potency for HCC treatment. The randomized, open-label, phase III REFLECT study revealed that lenvatinib is non-inferior to sorafenib in first-line treatment efficacy for patients with unresectable HCC [median OS: 13.6 months for lenvatinib *vs.* 12.3 months for sorafenib; hazard ratio (HR): 0.92; 95% confidence interval (CI): 0.79–1.06] (10). In addition, lenvatinib especially prolonged progression-free survival (PFS), time to progression (TTP), and objective response rate (ORR) compared with sorafenib (10). According to these positive results from the REFLECT study, lenvatinib therefore became the second approved agent in first-line systemic treatment for advanced HCC over the decade. Although lenvatinib and sorafenib both showed promising therapeutic efficacy, which systemic therapy should be firstly applied for patients with unresectable HCC is still of great concern in real clinical practice, so this study aimed to appraise therapeutic efficacy and safety of two orally administered first-line therapies, lenvatinib and sorafenib, for patients with unresectable HCC in the real world.

## Materials and Methods

### Patients

Patients with unresectable HCC in BCLC intermediate or advanced stages receiving lenvatinib or sorafenib in our institute, Kaohsiung Chang Gung Memorial Hospital, from January 2018 until August 2020 were enrolled. HCC was diagnosed by pathologic identification or dynamic imaging of abdominal computed tomography (CT) or magnetic resonance imaging (MRI) according to international HCC guidelines. The inclusion criteria were (1) unresectable HCC in BCLC intermediate or advanced stage; (2) receiving lenvatinib or sorafenib as first-line systemic therapy; and (3) classified as Child-Pugh (CP) class A or class B. Patients were excluded if they had previously received other systemic therapy; had insufficient clinical data; were concurrent with other cancers; were CP class C; or had become lost to follow-up during treatment. All included patients receiving lenvatinib or sorafenib further underwent propensity score (PS) matching to reduce the real-world baseline differences between Lenvatinib and Sorafenib groups. Data of the current study were retrospectively reviewed from the electronic medical charts and further analyzed. The study protocol was approved by the Research Ethics Committee of Chang Gung Memorial Hospital (IRB No: 202100961B0).

### Treatment Option

For unresectable HCC, using lenvatinib or sorafenib was based on the decision of clinicians and the wishes of patients. The dosage of lenvatinib or sorafenib was initially prescribed based on the recommendations of clinical trials (8, 10) and the experience of clinicians, and then was adjusted clinically by patients’ tolerance to the medication.

Patients in both groups received radiologic evaluation by CT or MRI every 2–3 months. Treatment with lenvatinib or sorafenib was stopped when tumor progression, liver function deterioration, intolerable treatment-related adverse events (TRAE), or death occurred. TRAE and disease progression were identified from the review of electronic medical records.

### Treatment Outcome

The outcomes of treatment were recorded that included PFS, defined as the time from treatment initiation to disease progression or death; TTP, defined as the time from treatment initiation to disease progression; OS, defined as the time from treatment initiation to death or the end of observation; ORR, defined as patients obtaining complete (CR) or partial response (PR); and DCR, defined as patients obtaining CR, PR, or stable disease status (SD). Treatment response was assessed by radiologic imaging according to the modified Response Evaluation Criteria in Solid Tumors (mRECIST) (11).

### Statistical Analysis

All patients were followed up till the date of last visit, death, or the end of April 2021. Comparing values between Lenvatinib and Sorafenib groups, Chi-squared tests were used for categorical variables, whereas Student’s *t*-test was applied for continuous variables. Quantitative variables were expressed with mean ± SD or median with a range. PS matching analyses were performed to minimize any selection biases and potential confounding variables. PS was calculated using logistic regression with the following variables: Age, Sex, Concentrations of α-fetoprotein (AFP), Child-Pugh score, HBV, HCV, BCLC stage, Extrahepatic Metastasis (EHM), and Macrovascular invasion (MVI), while for PS-matching analysis, a ratio of 1:2 matching scheme for lenvatinib *versus* sorafenib was applied. OS and PFS were assessed using the Kaplan-Meier method with a log-rank test, whereas univariate and multivariate analyses were estimated using Cox proportional hazards regression models. All *P*-values of <0.05 by two-tailed test were confirmed significant, with statistical analysis performed using SPSS 22 software (SPSS Inc., Chicago, IL, USA).

## Results

### Clinical Characteristics

A total of 322 patients including 81 (25.2%) with lenvatinib and 241 (74.8%) with sorafenib were further assigned to the Lenvatinib group (number, n=70) and the Sorafenib group (n=140) by using PS-matching analysis with a 1:2 ratio (**Supplementary Figure 1**). **Table 1** shows the characteristics of both groups before and after PS matching. Before PS matching, the Lenvatinib group had a significantly higher proportion of CP class B (8.6 *vs* 1.7%, p=0.003) and a lower proportion of dose-reduction patients (34.7 *vs* 88.7%, p<0.001) compared with the Sorafenib group. After the performance of PS matching, the baseline characteristics of the two groups were balanced, except that the proportion of dose reduction (37.1 *vs* 90.1%, p<0.001) remained lower in the Lenvatinib than in the Sorafenib group. In the PS-matched cohort, the mean age was 65.8 years and 70.4% of patients were male. Regarding the etiologies of HCC, 52.9% of patients were HBV infection and 26.7% were HCV infection. Additionally, 98.1% of patients were CP class A. Based on albumin-bilirubin (ALBI) scoring, there were 51.4% patients for ALBI grade I and 48.6% patients for grade II. The duration of drug use was not different between the two groups (4.9 months for lenvatinib-use *vs* 4.5 months for sorafenib-use, p=0.779).

**Table 1:** Baseline characteristics of the study population by treatment before and after propensity score matching.

Variables, n (%) or mean ± S.D.	Before Propensity Score Matching				After Propensity Score Matching^†^				
	Total N=322	Lenvatinib group N=81	Sorafenib group N=241	*P*-value	Total N=210		Lenvatinib group N=70	Sorafenib group N=140	*P*-value
Age (years)	63.7 ± 10.3	65.4 ± 11.9	65.1 ± 11.4	0.983	65.8 ± 11.5		65 ± 12.3	65.7 ± 11.6	0.983
Male Sex (%)	238 (73.9)	54 (66.7)	184 (76.3)	0.174	150 (71.4)		50 (71.4)	100 (71.4)	1
HCC etiology				0.221					0.672
HBV	165 (51.2)	38 (46.9)	127 (52.7)		111 (52.9)		36 (51.4)	75 (53.6)	
HCV	96 (29.8)	24 (29.6)	72 (29.9)		56 (26.7)		22 (31.4)	34 (24.3)	
Others	61 (19)	19 (23.5)	42 (17.4)		43 (20.4)		12 (17.2)	31 (22.1)	
Child-Pugh class				0.003					0.858
A	311 (96.6)	74 (91.4)	237 (98.3)		206 (98.1)		68 (97.1)	138 (98.6)	
B	11 (3.4)	7 (8.6)	4 (1.7)		4 (1.9)		2 (2.9)	2 (1.4)	
ALBI grade				0.672					0.558
I	156 (48.4)	38 (46.9)	118 (49)		108 (51.4)		34 (48.6)	74 (52.9)	
II	160 (49.7)	39 (48.1)	121 (50.2)		102 (48.6)		36 (51.4)	66 (47.1)	
III	6 (1.9)	2 (0.8)	2 (0.8)		0		0	0	
BCLC stage				0.167					0.707
B	59 (18.3)	19 (23.5)	40 (16.4)		39 (18.6)		14 (20)	25 (17.9)	
C	263 (81.7)	62 (76.5)	201 (83.4)		171 (81.4)		56 (80)	115 (82.1)	
AFP, ng/ml	8,137.3 ± 2053	8,515 ± 1,928	8,009 ± 2,097	0.853	7,707 ± 2,013		8,322 ± 1,978	7,396 ± 2,037	0.872
AFP ≥200 ng/ml	148 (46.1)	38 (46.9)	110 (45.8)	0.866	97 (46.2)		33 (47.1)	64 (45.7)	0.845
EHM	145 (45)	34 (42)	111 (46.1)	0.523	92 (43.8)		28 (40)	64 (45.7)	0.431
Lung	48 (14.9)	13 (16)	35 (14.5)		35 (16.7)		12 (17.1)	23 (16.4)	
Lymph node	59 (18.3)	13 (16)	46 (19.1)		33 (15.7)		10 (14.3)	23 (16.4)	
Bone	29 (9)	10 (12.3)	19 (7.9)		26 (12.4)		10 (14.3)	16 (11.4)	
MVI	161 (50)	34 (42)	127 (52.7)	0.085	95 (45.2)		33 (47.1)	62 (44.3)	0.695
VP3^‡^	97 (30.1)	21 (25.9)	76 (31.5)		60 (28.6)		20 (28.6)	40 (28.6)	
VP4^‡^	64 (19.9)	13 (16)	51 (21.2)		35 (16.7)		13 (18.6)	22 (15.7)	
Tumor size ≥6 cm,	107 (40.7)	29 (36.7)	78 (42.4)	0.39	60 (35.3)		25 (36.8)	35 (34.8)	0.743
Prior treatment	245 (77)	58 (71.6)	187 (78.9)	0.173	165 (78.9)		51 (72.9)	114 (82)	0.125
Surgery	82 (25.5)	22 (27.2)	60 (27.1)		56 (26.7)		20 (28.5)	36 (25.7)	
RFA	132 (41)	25 (30.1)	107 (48.4)		83 (39.5)		22 (31.4)	61 (43.6)	
TACE	181 (56.2)	36 (44.4)	145 (65.6)		120 (57.1)		31 (44.3)	89 (57.1)	
Combined treatment	82 (25.5)	22 (27.2)	60 (24.9)	0.686	57 (27.1)		18 (25.7)	39 (27.9)	0.742
Dose reduction	230 (75.4)	26 (34.7)	204 (88.7)	<0.001	154 (72.6)		26 (37.1)	128 (90.1)	<0.001
Duration of treatment	5.0 ± 4.6	5.1 ± 3.6	5.0 ± 4.9	0.927	4.6 ± 4		4.9 ± 3.6	4.5 ± 4.2	0.779

AFP, alpha fetoprotein; ALBI grade, albumin-bilirubin grade; BCLC, Barcelona Clinic Liver Cancer; EHM; extrahepatic metastasis; HBV, hepatitis B virus; HCC, hepatocellular carcinoma; HCV, hepatitis C virus; MVI, macrovascular invasion; RFA, radiofrequency ablation; TACE, transarterial chemoembolization.

^†^Propensity score was calculated using a 1:2 ratio-logistic regression with the following variables: **Age, Sex, BCLC stage, EHM, MVI, HBV, HCV, AFP, and Child-Pugh score.**

^‡^VP3: Tumor invasion into left portal vein or right portal vein; VP4: Tumor invasion into bilateral portal vein and/or main portal vein.

### Tumor Characteristics

After PS matching, 81.4% of patients had HCC in BCLC stage C, 45.2% of patients had tumors with MVI, and 43.8% of patients had EHM tumors respectively (**Table 1**). Among HCC patients with MVI, 36.8% were VP4 (tumor invasion into bilateral portal vein and/or main portal vein), whereas 63.2% were VP3 (tumor invasion into left or right portal vein). Concerning HCC patients with EHM, the top three spreading sites were the lung (38%), lymph node (35.8%), and bone (28.3%); additionally, 35.3% of patients had tumor burden larger than 6 cm in diameter.

### Prior and Combined Treatments

After PS matching, the proportion of patients who had received previous anti-HCC treatments was similar between Lenvatinib and Sorafenib groups (72.9 *vs* 82%, p=0.125). The leading three frequently used prior-treatment modalities were transarterial chemoembolization (TACE) (57.1%), radiofrequency ablation (RFA) (39.5%), and surgical resection (26.7%). Additionally, 25.7% of patients in the Lenvatinib group and 27.9% of patients in the Sorafenib group were treated with other combined treatments. The most two combination-used treatment modalities were TACE (n=23, 10.9%) and radiotherapy (n=18, 8.6%) respectively.

### Treatment Response in PS-Matched Cohort

After PS matching, 53 (75.7%) patients in the Lenvatinib group had follow-up dynamic images for the assessment of treatment response (**Table 2**). Among them, 1.9% of patients achieved CR, 7.5% obtained PR, 52.8% had SD, and 37.7% became progressive disease (PD). The ORR was 9.4%, whereas the DCR was 62.3%. The duration of lenvatinib durability was 7 months (range: 1.2–15 months). Concerning the Sorafenib group, among 111 patients (79.2%) with following dynamic images, 0.9% achieved CR, 7.2% obtained PR, 40.5% had SD, and 51.4% became PD.

**Table 2:** Tumor response by treatment^_†_^ in propensity score-matched cohort.

Variables, n (%) or median (range)	Lenvatinib group N=70	Sorafenib group N=140
Treatment response evaluation, n (%)	53 (75.7)	111 (79.2)
Complete response	1 (1.9)	1 (0.9)
Partial response	4 (7.5)	8 (7.2)
Stable disease	28 (52.8)	49 (40.5)
Progression disease	20 (37.7)	57 (51.4)
Objective response rate^‡^	9.4%	8.1%
Disease control rate^‡^	62.3%	48.6%
Durability, month	7 (1.2–15)	9.6 (1.0–24)
Death	28 (40)	91 (65)

^†^Treatment response based on those who received image evaluation including Computer tomography or Magnetic resonance image.

^‡^The comparison of objective response rate and disease control rate between two groups was 0.776 and 0.029, respectively.

### Progression-Free Survival and Its Associated Factors

**Figure 1:**
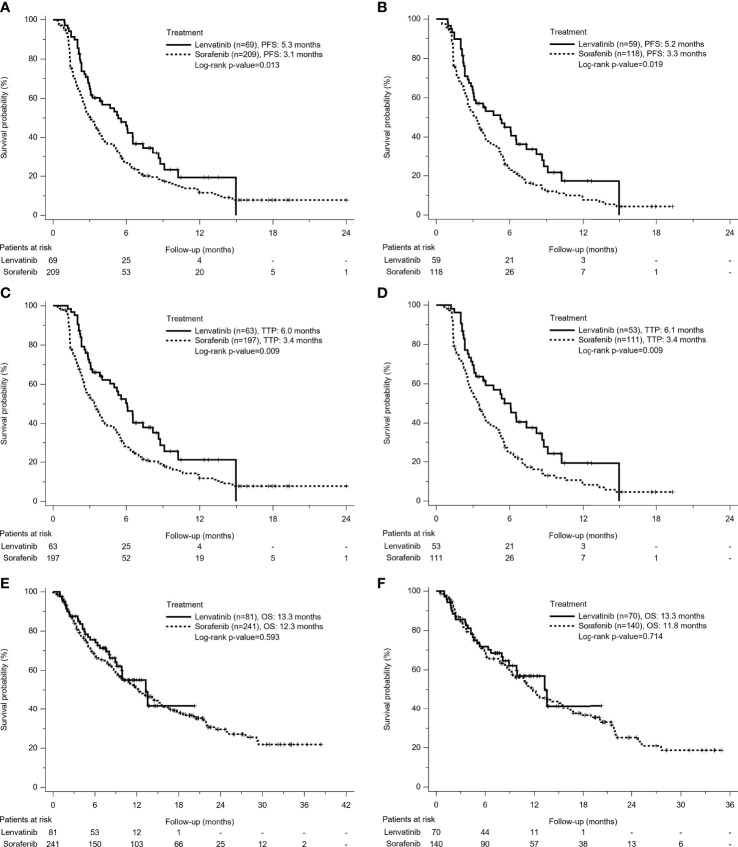
Kaplan-Meier survival curves of treatment outcomes including **(A)** Before Propensity Score (PS)-matched Progression-Free Survival (PFS), **(B)** After PS-matched PFS, **(C)** Before PS-matched Time to Progression (TTP), **(D)** After PS-matched TTP, **(E)** Before PS-matched Overall Survival (OS), and **(F)** After PS-matched OS between the Lenvatinib and Sorafenib groups. The Lenvatinib group had superior PFS and TTP, but similar OS to the Sorafenib group, no matter for either before or after PS matching analysis.

Among patients with radiologic evaluation, tumor progression was finally observed in 69.6% of the Lenvatinib group and 88% of the Sorafenib group. Patients treated with lenvatinib had significantly better PFS (5.3 *vs* 3.1 months, p=0.013) (**Figure 1A**) than patients treated with sorafenib. After PS matching, median PFS was still significantly longer in the Lenvatinib than in the Sorafenib group (5.2 months *vs* 3.3 months, p=0.019) (**Figure 1B**). Similarly, the Lenvatinib group also had better TTP than the Sorafenib group, whether before PS matching (6.0 *vs* 3.4 months, p=0.009) (**Figure 1C**) or after PS matching (6.1 *vs* 3.4 months, p=0.009) (**Figure 1D**). In Cox regression model of univariate and multivariate analyses, poorer liver function reserve, higher AFP level, and sorafenib use were independent risk factors associated with PFS in the PS-matched cohort (**Table 3**). Using lenvatinib could reduce the risk of progressing disease compared with using sorafenib (HR: 0.49, 95% CI: 0.3–0.79, p<0.001).

**Table 3:** Univariate and multivariate Cox regression analyses for Progression-Free Survival in propensity score-matched cohort.

Variables	Comparison	Univariate analysis		*P*-value	Multivariate analysis		*P*-value
		HR	95% CI		HR	95% CI	
Age, years	Increase per year	1.01	0.99–1.05	0.887			
Sex	Female *vs* Male	1.11	0.77–1.59	0.578			
HBV	Yes *vs* No	1.2	0.87–1.67	0.268			
HCV	Yes *vs* No	0.98	0.69–1.4	0.92			
ALBI Grade	II *vs* I	1.64	1.19–2.28	0.003	1.53	1.08–2.17	0.016
BCLC stage	C *vs* B	1.15	0.74–1.79	0.525			
EHM	Yes *vs* No	0.91	0.66–1.25	0.547			
MVI	Yes *vs* No	1.23	0.89–1.7	0.22			
AFP ≥200 ng/ml	Yes *vs* No	1.72	1.24–2.39	0.001	1.77	1.23–2.53	0.002
Dose reduction	Yes *vs* No	0.94	0.65–1.36	0.745			
Combined treatment	Yes *vs* No	0.77	0.54–1.1	0.156			
Treatment option	Lenvatinib *vs* Sorafenib	0.66	0.46–0.94	0.021	0.49	0.3–0.79	0.004

AFP, alpha fetoprotein; BCLC, Barcelona Clinic Liver Cancer; CI, confidence interval; EHM; extrahepatic metastasis; HBV, hepatitis B virus; HCV, hepatitis C virus; HR, hazard ratio; MVI, macrovascular invasion.

### Overall Survival and Its Associated Factors

A total of 181 patients (56.2%) died during the follow-up period, including 32 deaths (39.5%) in patients receiving lenvatinib and 149 deaths (61.8%) in patients receiving sorafenib. There was no difference of median OS in Lenvatinib and Sorafenib groups. (13.3 *vs* 12.3 months, p=0.593) (**Figure 1E**). After PS matching, median OS was 13.3 months in the Lenvatinib group and 11.8 months in the Sorafenib group, respectively (p=0.714) (**Figure 1F**). In multivariate analysis, poorer liver function reserve, higher AFP level, having no disease control, and no post-lenvatinib or sorafenib treatment were significant risk factors associated with mortality in PS-matched cohort (**Supplementary Table 1**). Different treatment agents using lenvatinib or sorafenib did not contribute to OS, whether for univariate or multivariate analysis.

### Treatment Safety in PS-Matched Cohort

After PS matching, the Lenvatinib group had higher proportions of total TRAE than the Sorafenib group (82 *vs* 75.9%, p=0.362), but there was no statistical difference (**Table 4**). Furthermore, the occurrence rate of severer TRAE (≥ grade 3) between the two groups was similar (11.5 *vs* 12%). In the Lenvatinib group, 82% of patients had incidence of TRAE, where the incidence over 9% included 26.2% of patients with hand-foot skin reaction (HFSR), 22.9% with hypertension, 19.7% with fatigue, and 9.6% with decreased appetite. Seven patients (11.5%) in the Lenvatinib group had severe TRAE over grade 3, and three of them were HFSR. Regarding the Sorafenib group, the top four TRAEs were HFSR (33.3%), diarrhea (25%), fatigue (13.9%), and decreased appetite (9.3%). Thirteen patients (12%) in the Sorafenib group developed grade 3 TRAE requiring treatment termination; similarly, HFSR was the most frequent TRAE (7 of 13).

**Table 4:** Treatment related adverse events (TRAE) by treatment in the propensity score-matched cohort.

Variables	Lenvatinib group (n=61)^†^		Sorafenib group (n=108)^†^	
	Any, n (%)	Grade ≥ 3, n (%)	Any, n (%)	Grade ≥ 3, n (%)
Total patients with TRAE	50 (82)	7 (11.5)	82 (75.9)	13 (12)
Hand foot skin reaction, n (%)	16 (26.2)	3 (4.8)	36 (33.3)	7 (6.3)
Hypertension, n (%)	14 (22.9)	1 (1.6)	4 (3.6)	0
Fatigue, n (%)	12 (19.7)	2 (3.2)	15 (13.9)	2 (1.8)
Diarrhea, n (%)	9 (14.8)	0	27 (25)	0
Decreased appetite, n (%)	6 (9.6)	0	10 (9.3)	0
Elevated T-bil, n (%)	3 (4.8)	0	2 (1.8)	1 (0.9)
Dysphonia, n (%)	3 (4.8)	0	0	0
Hypothyroidism, n (%)	2 (3.2)	0	0	0
Proteinuria, n (%)	2 (3.2)	0	0	0
Hepatic encephalopathy, n (%)	2 (3.2)	1 (1.6)	0	0
Pruritus, n (%)	2 (3.2)	0	2 (1.8)	0
Dermatitis, n (%)	1 (1.6)	0	3 (2.7)	1 (0.9)
Paresthesia, n (%)	0	0	1 (0.9)	1 (0.9)
UGI bleeding, n (%)	0	0	1 (0.9)	1 (0.9)

T-bil, total bilirubin; TRAE, treatment-related adverse event; UGI, upper gastrointestinal.

^†^Comparison of treatment-related adverse events was based on those patients who had medical records.

The comparison of any TRAE between two groups was 0.362.

### Subgroup Analysis for PFS in PS-Matched Cohort

**Figure 2:**
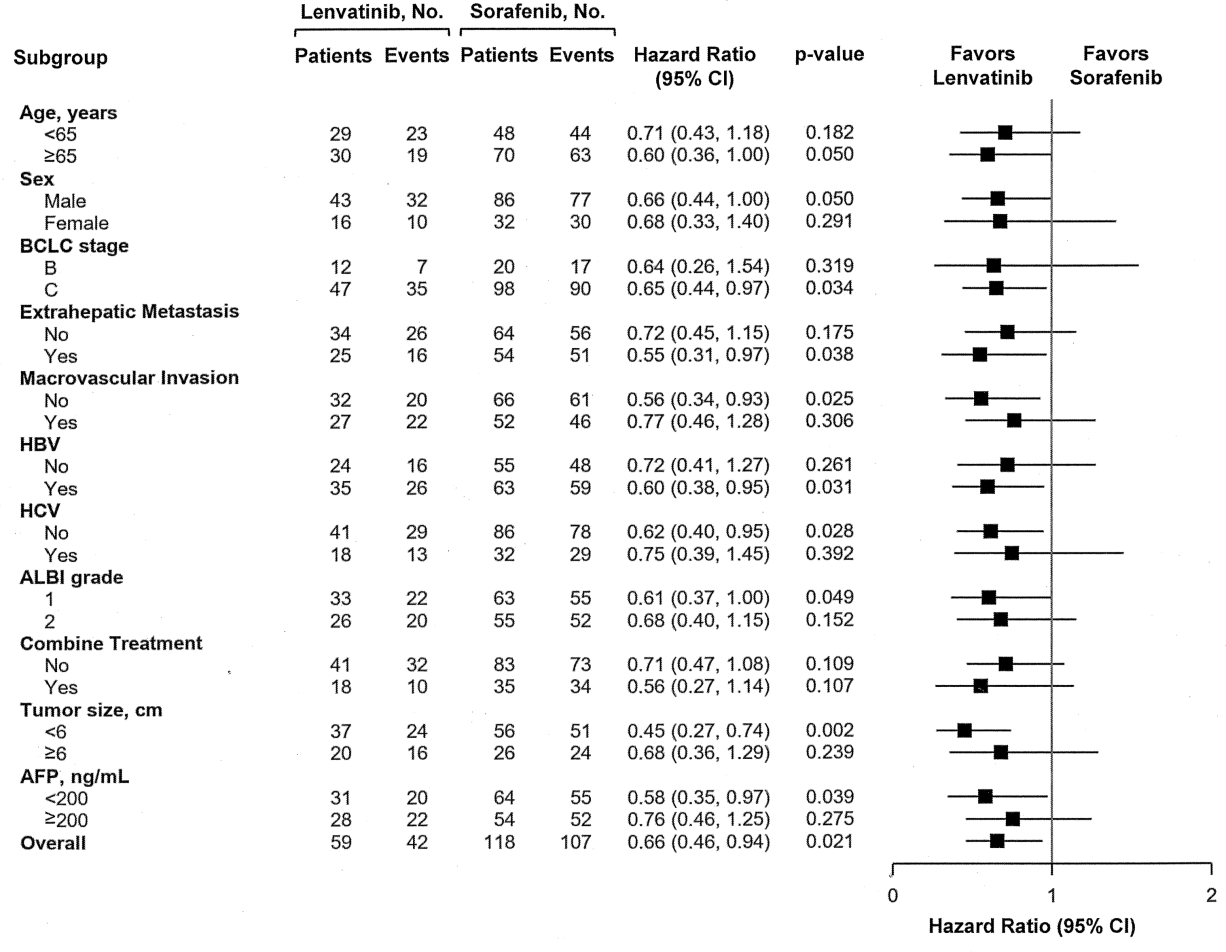
Forest plots of Progression-Free Survival in the subgroups of the Propensity Score-matched cohort.

After PS matching, the subgroup analysis indicated that using lenvatinib was superior or equal to using sorafenib associated with PFS in all subgroups (**Figure 2**). In particular, lenvatinib had a preferred role on better PFS for those patients in BCLC stage C, with HBV infection, without HCV infection, with EHM, without MVI, with tumor size <6 cm, or with AFP level <200 ng/ml. Overall, lenvatinib could reduce 34% of progression risk compared with sorafenib (p=0.021).

### Sequential Systemic Therapies After Treatment Failure in PS-Matched Cohort

After cessation of lenvatinib or sorafenib in PS-matched cohort, 103 patients (53%) still afforded following therapies (**Table 5**). Concerning sequential systemic therapies, 86 patients (44.3%) received second-line therapy, where regorafenib for 34 patients was the most frequently used agent followed in decreasing order by nivolumab for 16, lenvatinib for 13, chemotherapy for 12, atezolizumab plus bevacizumab for 5, carbozantinib for 3, and other agents for 3 patients. Twenty-one patients (10%) were treated with third-line therapy, whereas only two patients (0.9%) could move to fourth-line therapy.

**Table 5:** Sequential systemic treatments after failure of Lenvatinib or Sorafenib in the propensity score-matched cohort.

Variables	All, n=210	Lenvatinib, n=70	Sorafenib, n=140	P-value
Treatment Ongoing, n (%)	16 (7.6)	12 (17.1)	4 (2.9)	<0.001
Treatment Stop, n (%)	194 (92.4)	58 (82.9)	136 (97.1)	<0.001
Post-treatment, n (%)^†^	103 (53)	25 (43.1)	78 (57.4)	0.069
Second-line systemic treatment, n(%)^†^	86 (44.3)	21 (36.2)	65 (47.8)	0.02
Regorafenib	34	1	33	
Lenvatinib,	13	0	13	
Carbozantinib	3	2	1	
Nivolumab,	16	3	13	
Atezolizumab plus Bevacizumab	5	5	0	
Chemotherapy	12	9	3	
Others^‡^	3	1	2	
Third-line systemic therapy, n (%)	21 (10)	2 (3.4)	19 (14)	
Regorafenib	2	0	2	
Lenvatinib	5	0	5	
Nivolumab	8	2	6	
Pembrolizumab	2	0	2	
Chemotherapy	4	0	4	
Forth-line systemic therapy, n (%)	2 (0.9)	0 (0)	2 (1.5)	
Lenvatinib	1	0	1	
Nivolumab	1	0	1	

^†^The proportion was calculated in patients who stopped lenvatinib or sorafenib.

^‡^Others: Two patients received thalidomide and one patient received oral 5-Flurouracie, respectively.

## Discussion

Most of our patients used lenvatinib or sorafenib under reimbursement of the National Health Insurance (NHI) scheme of Taiwan (12), so that the baseline characteristics of this study population were relatively consistent. Additionally, although the current study was designed in retrospective setting, the analysis of 1:2 PS matching could reduce the select bias and confounding variables between Lenvatinib and Sorafenib groups in real life. Like the results of REFLECT trial (10), we also found significantly longer PFS in the Lenvatinib group (5.2 *vs* 3.3 months, p=0.019) but similar OR in two groups (13.3 *vs* 11.8 months, p=0.714). The reason of equivalent OS rates in Lenvatinib and Sorafenib groups might be due to different post-treatments offered, which possibly reduces the PFS difference between two groups. Almost all current post-treatment systemic agents were approved under the setting of following sorafenib failure (13–18), which leads to limited potential agents available for patients who failed lenvatinib, possibly inducing a disappointing post-treatment outcome.

Based on the design-setting of previous clinical trials (13, 14), only those patients who fail sorafenib remain CP class A liver function could apply for second-line therapies such as regorafenib or nivolumab by reimbursement of Taiwan NHI scheme. In the current study, 78 patients (57.4%) in the Sorafenib group and 25 patients (43.1%) in the Lenvatinib group could receive further anti-HCC treatments. Near half of the Sorafenib group (n=65, 47.8%) treated with second-line systemic therapies, including regorafenib (n=33) and nivolumab (n=13), were approved and reimbursed. On the contrary, in the Lenvatinib group, 21 patients (36.2%) received second-line systemic therapies where chemotherapy (n=9) under reimbursement was the main treatment modality, followed by self-paid agents including atezolizumab plus bevacizumab (n=5), nivolumab (n=3), carbozantinib (n=2), and regorafenib (n=1). In the real-world, high price of following multikinase inhibitors or immune-checkpoints (ICI) is really a great concern for those patients who fail lenvatinib but still have good liver function reserve. It usually keeps them from choosing these more effective but expensive agents. This could explain why sequential therapies after lenvatinib progression in the current study is not enough compared with sorafenib failure. For patients with the first-line lenvatinib treatment, lack of approved second-line treatments is a serious issue, but it is real in the present. More evidence-based studies regarding the efficacy and safety of sequential systemic therapies following lenvatinib are required to offer real-life data for the application or reimbursement of post-lenvatinib agents.

Regarding treatment response, we also indicate that using lenvatinib had a better DCR than using sorafenib (62.3 *vs* 48.6%, p=0.029); however, there was no significant difference in ORR between Lenvatinib and Sorafenib groups (9.4 *vs* 8.1%, p=0.776). Although there was an encouraging CR of 1.9%, and a PR of 7.5%, our ORR in the Lenvatinib group was lower than that in the REFLECT trial and some clinical studies (10, 19–21), in which it presented with a range from 14.1 to 29.6%. These differences might be due to different baseline liver function and tumor characteristics. In the comparison with the REFLECT trial, our Lenvatinib group had 18.6% of HCC patients with VP4 invasion and 20% with larger tumor size than 10 cm, presenting traditionally poorer tumor pictures of prognosis, possibly leading to a reduction of objective treatment response.

In the current study, most patients in the Lenvatinib or Sorafenib groups had good liver function status as CP class A and were suitable for treatment at the beginning (97.1 *vs* 98.6%, p=0.967). At the time of first-line treatment failure, 60.3% patients in the Lenvatinib group maintained Child A liver function, having the chance to receive following treatments, whereas 63.2% patients in the Sorafenib group still had Child A. The change of liver function during treatment was similar between both groups.

The current study indicates that better liver function reserve, lower concentration of AFP, achievement of disease control, and affording post-treatment were improved factors associated with mortality. However, using lenvatinib or sorafenib was not related to OS, no matter for either univariate or multivariate analysis. Despite there being no OS difference between Lenvatinib and Sorafenib groups, lenvatinib indeed improved the prognosis for patients with unresectable HCC.

In an era without effective sequential systemic therapies, our previous studies reported that the median OS of sorafenib use was only around 8 months (22, 23), whereas it was extended to 11.8 months in the current study. In general, near half of our study patients still required following therapies after failure of lenvatinib or sorafenib. Concerning sequential systemic therapies, 44.3% of patients received second-line therapy, where regorafenib was the most frequently used agent followed in decreasing order by nivolumab, lenvatinib, chemotherapy, atezolizumab plus bevacizumab, and carbozantinib. Approximately 10% of patients were treated with third-line therapy, whereas only a little under 1% of patients could move to fourth-line therapy.

In accordance with the REFLECT trial, the Lenvatinib group in our study could reduce the risk of tumor progression compared with the Sorafenib group. In multivariate analysis, lenvatinib was a significant predictor associated with PFS over sorafenib (HR:0.49, 95% CI: 0.3–0.79, p=0.004) after adjustment with ALBI grade and AFP concentration. Our finding was consistent with another real-life Korean study (24), in which performance status, AFP concentration, and lenvatinib *versus* sorafenib were associated factors with PFS. The REFLECT trial indicated that lenvatinib had better PFS than sorafenib in all subgroups, and we also found that lenvatinib was superior or equal to sorafenib on PFS in all subgroups. Particularly, for patients in BCLC stage C, with HBV infection, without HCV infection, with EHM, without MVI, with tumor size <6 cm or with AFP level <200 ng/ml, lenvatinib significantly prolonged PFS compared with sorafenib. Further subgroup studies are required to determine who the preferred target patients are for lenvatinib as first-line therapy.

With regard to treatment safety, the current study found that lenvatinib had similar incidences of overall TRAE compared with sorafenib (82 *vs* 75.9%, p=0.364). In fact, 37.1% of patients in our Lenvatinib group and 90.1% of patients in our Sorafenib group had experienced dosage reduction during treatment to maintain the balance of treatment efficacies and adverse events. Hence, occurrence rates of grade 3 TRAE between the two groups were equally low (11.5 *vs* 12%). Interestingly, the most frequent adverse event in our study was HFSR (26.2%), reported as having lower incidence compared with hypertension or diarrhea in previous clinical studies (20, 21, 25, 26). Actually, the incidence of hypertension (22.9%) in the current study was close to that of HFSR but might be underestimated, because some clinicians were initially not familiar with this TRAE and records of blood pressure were lacking.

The current study still has some limitations. Firstly, some clinical and laboratory data were not available from medical chart review due to retrospective setting of this study. Approximately 22% of the patients were short of image examinations following treatment, and this might influence evaluation of treatment response; additionally, incidence of some TRAEs might be underestimated. Secondly, sequential systemic therapies are not established for patients who failed first-line lenvatinib. Hence, the second-line systemic therapies following lenvatinib or sorafenib are not consistent, which might generate confounding bias in analysis of post-treatment survival. Thirdly, lenvatinib has been reimbursed by the NHI of Taiwan since 2019, so that most patients in the Lenvatinib group had shorter observation time than in the Sorafenib group. Further longer follow-up duration might provide a clearer picture of treatment outcome in real life. Finally, the current study is a single-center retrospective study with limited sample size; further multicenter validated studies are required.

In conclusion, the current study demonstrates that lenvatinib is an appropriate first-line therapy for unresectable HCC with promising survival effects and tolerable adverse events in clinical real practice. Compared with sorafenib, lenvatinib did not extend overall survival, but it could really improve treatment response and reduce the risk of disease progression. Additionally, lack of approved post-lenvatinib systemic treatments is a severe issue in the real world.

## Data Availability Statement

The original contributions presented in the study are included in the article/**Supplementary Material**. Further inquiries can be directed to the corresponding author.

## Ethics Statement

The studies involving human participants were reviewed and approved by the Institutional Review Board of our institute: Kaohsiung Chang Gung Memorial Hospital (IRB No: 202100961B0). Written informed consent for participation was not required for this study in accordance with the national legislation and the institutional requirements.

## Author Contributions

Y-HK and J-HW made substantial contributions to the study conception, design, analysis, and interpretation of the data. S-NL, Y-YC, K-MK, Y-HY, C-HH, T-HH, and C-HC contributed to the acquisition of the data. The first draft of the manuscript was written by Y-HK and J-HW. J-HW commented on subsequent versions of the manuscript. All authors contributed to the article and approved the submitted version.

## Conflict of Interest

The authors declare that the research was conducted in the absence of any commercial or financial relationships that could be construed as a potential conflict of interest.

## Publisher’s Note

All claims expressed in this article are solely those of the authors and do not necessarily represent those of their affiliated organizations, or those of the publisher, the editors and the reviewers. Any product that may be evaluated in this article, or claim that may be made by its manufacturer, is not guaranteed or endorsed by the publisher.

## Supplementary Material

Supplementary Figure 1 Flow chart of the study population. Propensity Score Matching was performed with a 1:2 ratio.
