# Corrigendum: Real-World Lenvatinib *Versus* Sorafenib in Patients With Advanced Hepatocellular Carcinoma: A Propensity Score Matching Analysis

**DOI:** 10.3389/fonc.2021.823960

**Published:** 2021-12-14

**Authors:** Yuan-Hung Kuo, Sheng-Nan Lu, Yen-Yang Chen, Kwong-Ming Kee, Yi-Hao Yen, Chao-Hung Hung, Tsung-Hui Hu, Chien-Hung Chen, Jing-Houng Wang

**Affiliations:** ^1^ Division of Hepatogastroenterology, Department of Internal Medicine, Kaohsiung Chang Gung Memorial Hospital and Chang Gung University College of Medicine, Kaohsiung, Taiwan; ^2^ Division of Hematology-Oncology, Department of Internal Medicine, Kaohsiung Chang Gung Memorial Hospital and Chang Gung University College of Medicine, Kaohsiung, Taiwan

**Keywords:** hepatocellular carcinoma, lenvatinib, propensity score (PS) matching (PSM), sorafenib, progression-free survival

In the original article, there was an error. **The IRB Number of our article, “IRB No: 202100961B0”, was typed mistakenly as “IRB No: 20210096B0”.**

A correction has been made to the last sentence of the ***Materials and Methods***, *Patients* and the ***Ethics Statement***”.

The authors apologize for this error and state that this does not change the scientific conclusions of the article in any way. The original article has been updated.

## Publisher’s Note

All claims expressed in this article are solely those of the authors and do not necessarily represent those of their affiliated organizations, or those of the publisher, the editors and the reviewers. Any product that may be evaluated in this article, or claim that may be made by its manufacturer, is not guaranteed or endorsed by the publisher.

