# Supplementary material for: Real-World Lenvatinib Versus Sorafenib in Patients With Advanced Hepatocellular Carcinoma: A Propensity Score Matching Analysis
Source: Front Oncol. 2021 Oct 25;11:737767. doi: 10.3389/fonc.2021.737767 (PMC8573180; doi:10.3389/fonc.2021.737767)
Supplement: Supplementary file 2 [file Table_1.docx]

Table 1. Uni-variate and multi-variate cox regression analysis for Overall Survival in propensity score-matched cohort

| Variables | Comparison | Univariate analysis | |  | Multivariate analysis | |  |
| --- | --- | --- | --- | --- | --- | --- | --- |
|  |  | H.R | 95% CI | *P*-value | H.R | 95% CI | *P*-value |
| Age, years | Increase per year | 1.00 | 0.99-1.02 | 0.607 |  |  |  |
| Sex | Female vs. Male | 1.24 | 0.84-1.83 | 0.277 |  |  |  |
| HBV | Yes vs. No | 1.04 | 0.77-1.44 | 0.982 |  |  |  |
| HCV | Yes vs No | 0.96 | 0.65-1.43 | 0.848 |  |  |  |
| ALBI Grade | II vs I | 2.44 | 1.69-3.52 | <0.001 | 1.67 | 1.03-2.66 | 0.037 |
| BCLC stage | C vs B | 1.61 | 0.96-2.69 | 0.07 |  |  |  |
| EHM | Yes vs No | 0.9 | 0.63-1.3 | 0.578 |  |  |  |
| MVI | Yes vs No | 1.51 | 1.05-2.16 | 0.027 |  |  |  |
| AFP ≥ 200 ng/ml | Yes vs No | 2.81 | 1.93-4.08 | <0.001 | 2.04 | 1.31-3.17 | 0.002 |
| Disease control | Yes vs No | 0.37 | 0.24-0.56 | <0.001 | 0.34 | 0.21-0.55 | <0.001 |
| Combined treatment | Yes vs No | 0.76 | 0.51-1.16 | 0.202 |  |  |  |
| Post treatment | Yes vs No | 0.26 | 0.18-0.39 | <0.001 | 0.32 | 0.19-0.56 | <0.001 |
| Treatment option | Lenvatinib vs. Sorafenib | 0.92 | 0.59-1.43 | 0.714 |  |  |  |

Abbrevations: AFP, alpha fetoprotein; BCLC, Barcellola Clinic Liver Cancer; CI, confidence interval; EHM; extra-hepatic metastasis; HBV, hepatitis B virus; HCV, hepatitis C virus; HR, hazard ratio; MVI, macro-vascular invasion.
